# A Vascular Invasion-Related Gene Signature Identifies NUP35 as a Driver of Angiogenesis and Poor Prognosis in Pancreatic Ductal Adenocarcinoma

**DOI:** 10.3390/biomedicines14061253

**Published:** 2026-05-30

**Authors:** Hong Sun, Haiqing Tian, Lei Feng, Wuhao Ying, Yikai Wang, Song Gu, Weihong Xu, Jun Chu

**Affiliations:** 1 Department of Anesthesiology, Shanghai Children’s Medical Center, Shanghai Jiao Tong University School of Medicine, Shanghai 200127, China; 2 Department of General Surgery, Shanghai Children’s Medical Center, Shanghai Jiao Tong University School of Medicine, Shanghai 200127, China

**Keywords:** pancreatic ductal adenocarcinoma, vascular invasion, prognostic signature, NUP35, angiogenesis

## Abstract

**Background**: Pancreatic ductal adenocarcinoma (PDAC) has dismal survival, and vascular invasion is strongly associated with dissemination and poor outcomes; however, its molecular basis remains unclear. **Methods**: Transcriptomic and clinical data from The Cancer Genome Atlas-Pancreatic Adenocarcinoma (TCGA-PAAD) cohort were integrated with Genotype-Tissue Expression (GTEx) normal pancreas data. Vascular invasion-associated candidate genes were identified using subgroup-specific differential expression filtering. A three-gene prognostic signature was constructed using Cox and least absolute shrinkage and selection operator (LASSO) regression and validated in an independent PACA-AU cohort. Nucleoporin 35 (NUP35) was functionally evaluated by shRNA knockdown, phenotypic assays, endothelial assays using conditioned medium, and Western blotting of extracellular signal-regulated kinase (ERK)-vascular endothelial growth factor A (VEGFA) signaling. **Results**: We identified 172 vascular invasion-associated candidate genes and developed a three-gene model comprising NUP35, GMNN, and KLK11. The model stratified TCGA patients into risk groups with significantly different overall survival (OS; log-rank *p* = 0.014) and good predictive performance, with areas under the receiver operating characteristic curve (AUC) of 0.659, 0.722, and 0.796 for 1-, 3-, and 5-year OS, respectively. Consistent trends were observed in PACA-AU. Risk group transcriptomes were enriched for proliferative and tumor progression programs. Among the signature genes, NUP35 was prioritized because higher NUP35 expression was associated with poorer survival and positively correlated with VEGFA expression. NUP35 knockdown suppressed malignant phenotypes, reduced endothelial migration and tube formation, and decreased phosphorylated ERK and VEGFA without altering total ERK levels. **Conclusions**: A vascular invasion-related three-gene signature enables prognostic stratification in PDAC. NUP35 is associated with malignant and pro-angiogenic phenotypes and may regulate angiogenic activity partly through ERK-VEGFA signaling, supporting its potential as a prognostic biomarker and candidate therapeutic vulnerability.

## 1. Introduction

Pancreatic ductal adenocarcinoma (PDAC) is among the most lethal malignancies worldwide, characterized by aggressive biological behavior, early metastatic spread, and dismal prognosis [1,2]. Despite advances in surgical techniques and systemic therapies, long-term survival remains poor for most patients with PDAC [3]. Notably, surgical resection remains the only potentially curative treatment, but only approximately 15–20% of patients present with resectable disease at diagnosis [3]. Treatment strategies are therefore largely stage-dependent and include surgery-based multimodality treatment for resectable disease and systemic therapy for advanced stage disease [3,4]. Among the pathological features associated with tumor aggressiveness, vascular invasion is of particular clinical importance because it facilitates hematogenous dissemination and is closely associated with advanced stage and unfavorable outcomes [5,6]. However, the molecular basis of vascular invasion in PDAC remains incompletely understood [7], which limits its integration into prognostic evaluation and therapeutic decision-making.

Angiogenesis is a key process in tumor progression and vascular invasion, and is driven, at least in part, by pro-angiogenic mediators such as vascular endothelial growth factor A (VEGFA) [8,9]. Although angiogenesis has been extensively investigated in PDAC, anti-angiogenic therapies have yielded limited clinical benefit [10,11], indicating that additional molecular programs may regulate tumor-vascular crosstalk. With the development of high-throughput sequencing and bioinformatics approaches, it is now possible to systematically identify molecular signatures linked to aggressive tumor phenotypes [12]. Nevertheless, most currently available prognostic models for PDAC are not specifically designed around vascular invasion-related features, and many proposed candidate genes lack experimental validation.

In this study, we systematically analyzed vascular invasion-associated transcriptional alterations in PDAC and developed a vascular invasion-related prognostic signature through survival-based gene selection and regularized Cox regression. We then evaluated its prognostic performance in independent cohorts and further prioritized a key candidate gene for functional validation based on its clinical relevance and association with angiogenesis-related features. Collectively, our findings provide insight into the molecular landscape of vascular invasion in PDAC and support the potential utility of vascular invasion-related biomarkers for prognostic stratification and therapeutic exploration.

## 2. Materials and Methods

### 2.1. Data Acquisition and Processing

Transcriptomic and clinical data for PDAC were obtained from The Cancer Genome Atlas (TCGA; TCGA-PAAD). Gene expression profiles and corresponding clinical annotations were downloaded from the Genomic Data Commons (GDC) using the R package TCGAbiolinks. Because TCGA-PAAD contains a limited number of normal pancreatic samples, normal pancreas expression data in fragments per kilobase of transcript per million mapped reads (FPKM) format were retrieved from the Genotype-Tissue Expression (GTEx) project through the University of California Santa Cruz (UCSC) Xena platform and incorporated as non-tumor controls for tumor normal differential expression analyses. Batch effects between TCGA and GTEx datasets were corrected using the ComBat algorithm implemented in the sva R package. Expression values were transformed as log_2_(FPKM + 1) prior to downstream analyses.

### 2.2. Identification of Vascular Invasion-Associated Candidate Genes in PDAC

PDAC samples were stratified into vascular invasion-positive and vascular invasion-negative groups according to the angiolymphatic invasion annotation in TCGA-PAAD pathology records. Samples with documented angiolymphatic invasion were classified as vascular invasion-positive, whereas those without documented invasion were classified as vascular invasion-negative. Samples with missing or indeterminate annotations were excluded from vascular invasion-based grouping analyses. Because TCGA-PAAD samples are heterogeneous with respect to tumor purity, stromal composition, stage distribution, and clinical background [13], direct comparison between vascular invasion-positive and vascular invasion-negative tumor tissues may yield limited or unstable differential signals. Therefore, to reduce the influence of shared PDAC-related transcriptional alterations, we used a predefined subgroup-specific tumor-vs-normal filtering strategy. Differential expression analyses were performed separately by comparing vascular invasion-positive tumors vs. normal pancreas and vascular invasion-negative tumors vs. normal pancreas, and the corresponding results are provided in Supplementary Data S1 and S2, respectively. Differential expression was assessed using the Wilcoxon rank-sum test, with *p* values adjusted using the Benjamini–Hochberg method. Genes with |log_2_ fold change| > 1 and false discovery rate (FDR) < 0.05 were considered differentially expressed genes (DEGs). Vascular invasion-associated candidate genes were defined using subgroup-specific filtering. Genes uniquely upregulated in the vascular invasion-positive comparison (but not significant in the vascular invasion-negative comparison) and genes uniquely downregulated in the vascular invasion-negative comparison (but not significant in the vascular invasion-positive comparison) were retained for subsequent analyses.

### 2.3. Functional Enrichment Analysis

Gene Ontology (GO) enrichment analysis, including biological process (BP), cellular component (CC), and molecular function (MF) categories, was performed in R using clusterProfiler (version 4.16.0), org.Hs.eg.db (version 3.21.0), and enrichplot (version 1.28.4). Kyoto Encyclopedia of Genes and Genomes (KEGG) enrichment analysis was conducted using the Kyoto Encyclopedia of Genes and Genomes Orthology-Based Annotation System (KOBAS) web server (http://bioinfo.org/kobas/) (accessed on 26 May 2026). Integrated enrichment analysis was additionally performed using Metascape, including GO, KEGG, and Reactome terms. Terms and pathways with adjusted *p* < 0.05 were considered significant.

To further address angiogenesis-related transcriptional changes in the vascular invasion-negative phenotype, angiogenesis-related genes were defined using the MSigDB Hallmark Angiogenesis gene set (https://www.gseamsigdb.org/gsea/msigdb/cards/HALLMARK_ANGIOGENESIS) (accessed on 26 May 2026) and intersected with DEGs identified in the vascular invasion-negative vs. normal pancreas comparison. The resulting genes are summarized in Supplementary Data S4.

### 2.4. Development and Validation of the Vascular Invasion-Related Prognostic Signature

Univariate Cox proportional hazards regression was performed in TCGA-PAAD to identify vascular invasion-associated genes significantly associated with overall survival (OS) (*p* < 0.05). Least absolute shrinkage and selection operator (LASSO) Cox regression was then applied using glmnet R package (version 4.1.10) with tenfold cross-validation to select robust prognostic markers [14]. A multivariate Cox proportional hazards model was fitted using the selected genes and coefficients from the multivariate Cox model were used to calculate the risk score. The risk score for each patient was calculated using gene expression levels weighted by the corresponding Cox regression coefficients according to the following formula: Risk score = Σ (Coef_i_ × Expression_i_), i = 1 … n, where Coef_i_ represents the regression coefficient of gene i, Expression_i_ represents the expression level of gene i, and n represents the number of signature genes.

For model evaluation in TCGA, patients were stratified into high- and low-risk groups using the median risk score as the cutoff. Prognostic performance was evaluated by Kaplan–Meier survival analysis (log-rank test), time-dependent receiver operating characteristic (ROC) analysis using timeROC R package (version 0.4.1), and visualization of risk distribution and survival status using ggrisk R package (version 1.3). A nomogram incorporating the final prognostic signature was constructed using the rms R package (version 8.1.1). To evaluate whether the prognostic signature provided prognostic information independent of available clinicopathological variables, multivariable Cox regression analysis was performed using the risk score and clinical covariates, including age, gender, and pathological stage. Because only a small number of patients were classified as stage III/IV, pathological stage was collapsed into stage I and stage II–IV groups to avoid model instability caused by sparse events in detailed stage subgroups. External validation was performed in an independent PDAC cohort from the International Cancer Genome Consortium (ICGC; PACA-AU). Risk scores were calculated using coefficients derived from the TCGA multivariate Cox model and patients were classified into high- and low-risk groups based on the median risk score within the validation cohort.

### 2.5. Transcriptomic Analyses Based on Risk Score and Prioritized Candidate Gene Expression

For transcriptome-level contrast analyses, including volcano plots, enrichment analyses and gene set enrichment analysis (GSEA), samples were grouped using a quartile-based strategy to enhance biological contrast. Differential expression analyses used the same criteria described above (Wilcoxon rank-sum test; |log_2_ fold change| > 1; FDR < 0.05). GSEA was performed using GSEA desktop software (v4.3.2; Broad Institute, Cambridge, MA, USA) in preranked mode with ranked gene lists derived from differential expression results. Enrichment was reported as normalized enrichment score (NES) with FDR-adjusted *p* values.

### 2.6. Survival and Correlation Analyses

After construction of the prognostic model, survival analyses for the final signature genes were performed across TCGA and ICGC datasets using the Biomarker Exploration of Solid Tumors (BEST) online platform (https://rookieutopia.hiplot.com.cn/app_direct/BEST/) (accessed on 26 May 2026), reporting Kaplan–Meier curves, hazard ratios (HRs), and log-rank *p* values for available endpoints (OS, progression-free survival (PFS), or relapse-free survival (RFS)). Correlation and pan-cancer analyses were conducted using TIMER2.0 (https://compbio.cn/timer2/) (accessed on 26 May 2026) and TCGAplot R package (version 7.0.1), including correlations with VEGFA in PDAC, associations with angiogenesis-related genes and signatures, tumor mutation burden (TMB), microsatellite instability (MSI), immune checkpoint molecules, and immune cell infiltration estimates. Spearman’s rank correlation, or Pearson’s correlation where specified, was used, and statistical significance followed platform-implemented methods with multiple-testing correction when available.

### 2.7. Human Protein Atlas Analysis

Nucleoporin 35 (NUP35) protein expression and subcellular localization were assessed using the Human Protein Atlas (HPA, https://www.proteinatlas.org) (accessed on 26 May 2026). HPA immunohistochemistry (IHC) images were used to compare NUP35 protein expression between pancreatic cancer and normal pancreatic tissues. In addition, HPA immunofluorescence (IF) images were reviewed to evaluate the subcellular localization of NUP35.

### 2.8. Cell Culture and Generation of NUP35 Knockdown Cells

PATU 8988 human pancreatic cancer cells, HEK293T cells and human umbilical vein endothelial cells (HUVECs) were obtained from Shanghai Fuheng Biotechnology Co., Ltd. (Shanghai, China). PATU 8988 and HEK293T cells were cultured in Dulbecco’s modified Eagle’s medium (DMEM) (G4515, Servicebio, Wuhan, China) supplemented with 10% fetal bovine serum (FBS) (G8003, Servicebio) and 1% penicillin–streptomycin (C0222, Beyotime, Shanghai, China) at 37 °C in a humidified incubator with 5% CO_2_. HEK293T cells were used for lentiviral packaging. HUVECs were maintained in endothelial cell growth medium (FHS06, Shanghai Fuheng Biotechnology) under standard conditions (37 °C, 5% CO_2_).

Two short hairpin RNAs (shRNAs) targeting NUP35 (sh-1, GACTCCACAACCTCGATCAAT; sh-2, GCTCCACCAGTTAGAAGTATA) were cloned into the pLKO.1 lentiviral vector obtained from MiaoLing Plasmid Platform (Wuhan, China). Lentiviral particles were produced in HEK293T cells by cotransfection of pLKO.1-shRNA, psPAX2, and pMD2.G plasmids, all provided by MiaoLing Plasmid Platform, at a plasmid ratio of 5:4:1 using the Lipo8000 transfection reagent (C0533, Beyotime). Viral supernatants were collected at 48 and 72 h post-transfection and concentrated using 100 kDa ultrafiltration units (FUF390, Beyotime). PATU 8988 cells were infected with concentrated lentivirus and selected using puromycin (2 μg/mL) (ST551, Beyotime) to establish stable knockdown lines. A non-targeting shRNA served as the negative control (sh-NC).

### 2.9. Western Blot Analysis

Cells were lysed in radioimmunoprecipitation assay (RIPA) buffer (P0013B, Beyotime) supplemented with protease and phosphatase inhibitors (P1045, Beyotime), and protein concentrations were determined using a BCA Protein Assay Kit (P0010, Beyotime). Protein (20 μg) was separated by SDS-PAGE and transferred onto polyvinylidene fluoride (PVDF) membranes (IPVH00010, Merck Millipore, Billerica, MA, USA). Membranes were incubated with primary antibodies against NUP35 (A12762, ABclonal, Wuhan, China), VEGFA (A12303SP, ABclonal), extracellular signal-regulated kinase (ERK, A4782, ABclonal), phospho-ERK (p-ERK, AP0974, ABclonal), and α-tubulin (A6830, ABclonal), followed by HRP-conjugated secondary antibodies (AS014, ABclonal). Bands were visualized by enhanced chemiluminescence (ECL) (P0018S, Beyotime) and imaged using a Bio-Rad ChemiDoc MP system (Bio-Rad Laboratories, Hercules, CA, USA). Band intensities were quantified using ImageJ (v1.53t; National Institutes of Health, Bethesda, MD, USA), and p-ERK and VEGFA levels were normalized to α-tubulin. Experiments were performed with at least three independent biological replicates.

### 2.10. Functional Assays

For wound healing assays, PATU 8988 cells (sh-NC, sh-1, and sh-2) were grown to 90–100% confluence in six-well plates, scratched using a sterile 200 μL pipette tip, and cultured in serum-free DMEM (G4515, Servicebio). Images were acquired at 0, 24, and 48 h, and wound closure was quantified using ImageJ.

Cell proliferation was assessed using a 5-ethynyl-2′-deoxyuridine (EdU) incorporation assay (BeyoClick™ EdU Cell Proliferation Kit with Alexa Fluor 594, C0078S, Beyotime). Cells were seeded at 2 × 10^3^ cells/well in 96-well plates, labeled with 10 μM EdU for 2 h at 37 °C, and processed according to the manufacturer’s instructions. The EdU-positive rate was calculated as EdU-positive nuclei divided by total Hoechst 33342 (C1026, Beyotime)-stained nuclei across at least five random fields per well.

Migration and invasion were evaluated using 24-well transwell chambers with 8 μm pores (Corning Inc., Corning, NY, USA). For migration assays, 5 × 10^4^ cells were seeded in serum-free medium (G4515, Servicebio) and incubated for 24 h. For invasion assays, inserts were precoated with Matrigel (C0372, Beyotime), and 1 × 10^5^ cells were incubated for 48 h. Cells were fixed with 4% paraformaldehyde (P0099, Beyotime) and stained with 0.1% crystal violet (C0121, Beyotime). Images were captured under a light microscope (IX73, Olympus, Tokyo, Japan), and migrated or invaded cells were manually counted in five randomly selected fields per insert. The mean cell number from the five fields was used for statistical analysis.

### 2.11. Endothelial Migration and Tube Formation Assays Using Conditioned Medium

Conditioned medium (CM) was collected from sh-NC, sh-1, and sh-2 PDAC cells. When tumor cells reached ~70–80% confluence, the medium was replaced with serum-free DMEM (G4515, Servicebio) for 24 h. Supernatants were collected, centrifuged to remove debris, filtered through a 0.22 μm membrane (SLGPR33RB, Merck Millipore), and mixed 1:1 with complete endothelial medium (FHS06, Shanghai Fuheng Biotechnology) for downstream assays.

For endothelial migration, confluent HUVEC monolayers were scratched using a sterile 200 μL pipette tip and then cultured in CM. Images were captured at 0, 12, and 24 h, and wound closure was quantified. For tube formation, Matrigel (50 μL/well, C0372, Beyotime) was polymerized in pre-chilled 96-well plates at 37 °C for 60 min. HUVECs (1 × 10^4^ cells/well) were seeded in CM and incubated for 8 h. Tube-like networks were imaged under a bright-field microscope (IX73, Olympus), and branch points and capillary length (μm/field) were quantified using ImageJ (v1.53t; National Institutes of Health) with the Angiogenesis Analyzer plugin.

### 2.12. Statistical Analysis

Statistical analyses were performed using R (version 4.2.0) and GraphPad Prism (version 9.0, GraphPad Software, San Diego, CA, USA). Data are presented as mean ± standard deviation (SD) from at least three independent biological replicates unless otherwise specified. Two-group comparisons used a two-tailed Student’s t test or Wilcoxon rank-sum test, as appropriate. Comparisons among three groups were performed using one-way analysis of variance (ANOVA) followed by Tukey’s post hoc test for normally distributed data. Otherwise, nonparametric alternatives were applied. Survival analyses used the Kaplan–Meier method and log-rank test, and Cox regression was used for prognostic evaluation. Multiple testing in high-dimensional analyses was controlled using the Benjamini–Hochberg method (FDR < 0.05). Otherwise, two-sided *p* < 0.05 was considered statistically significant.

## 3. Results

### 3.1. Clinicopathological Characteristics of Pancreatic Ductal Adenocarcinoma Patients Stratified by Vascular Invasion Status

A total of 164 patients with PDAC from the TCGA cohort were included and stratified according to vascular invasion status (vascular invasion-positive, *n* = 104; vascular invasion-negative, *n* = 60). The clinicopathological characteristics of the two groups are summarized in Table 1.

Tumor stage, T, N, and M were defined according to the American Joint Committee on Cancer (AJCC) tumor-node-metastasis (TNM) staging system, where T stage indicates primary tumor extent, N stage indicates regional lymph node involvement, and M stage indicates distant metastasis status [15]. No significant differences were observed between the two groups in age, sex, race, tumor size, M stage, tumor grade, tumor location, or Kirsten rat sarcoma viral oncogene homolog (KRAS) mutation status (all *p* > 0.05). In contrast, vascular invasion status was significantly associated with overall tumor stage (*p* = 0.0311), T stage (*p* = 0.0189), and N stage (*p* < 0.0001), indicating that vascular invasion is closely linked to more advanced disease in PDAC.

### 3.2. Identification of Vascular Invasion-Associated Candidate Genes in Pancreatic Ductal Adenocarcinoma

**Figure 1:**
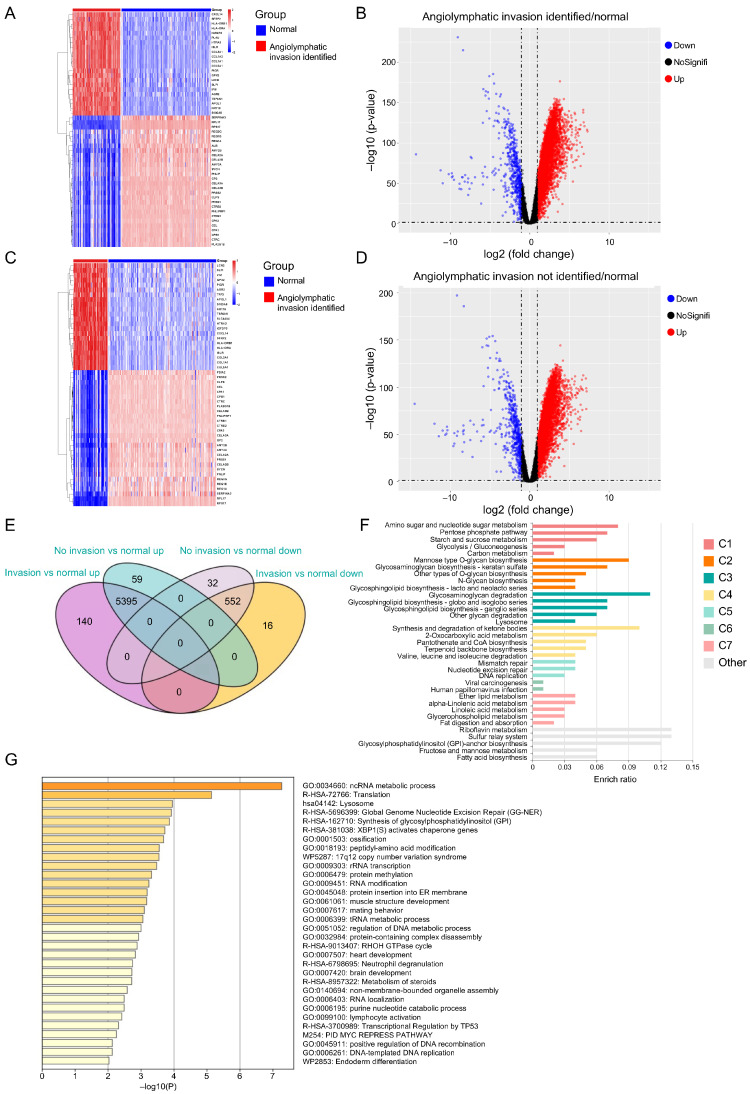
Identification and functional annotation of vascular invasion-associated genes in PDAC. (**A**) Heatmap showing representative top DEGs between vascular invasion-positive PDAC tissues and normal pancreatic tissues. (**B**) Volcano plot of DEGs in vascular invasion-positive PDAC tissues vs. normal controls. (**C**) Heatmap showing representative top DEGs between vascular invasion-negative PDAC tissues and normal pancreatic tissues. (**D**) Volcano plot of DEGs in vascular invasion-negative PDAC tissues vs. normal controls. In the volcano plots, “Up”, “Down”, and “NoSignifi” indicate significantly upregulated, significantly downregulated, and nonsignificant genes, respectively. (**E**) Venn diagram illustrating overlaps between DEG sets and subgroup-specific PDAC genes used to define vascular invasion-associated genes. “Invasion” and “No invasion” indicate angiolymphatic invasion identified and not identified in PDAC tissues compared with normal pancreas, respectively. (**F**) KEGG pathway enrichment analysis of vascular invasion-associated genes (KOBAS). Enrich ratio indicates the proportion of input genes mapped to each KEGG pathway. (**G**) Metascape integrated enrichment analysis of vascular invasion-associated genes.

To identify genes associated with vascular invasion in PDAC, we performed differential expression analyses by comparing vascular invasion-positive PDAC tissues vs. normal pancreatic tissues and vascular invasion-negative PDAC tissues vs. normal pancreatic tissues. As shown in Figure 1A,B, vascular invasion-positive PDAC samples displayed a distinct transcriptomic profile relative to normal controls with clear separation in the heatmap and numerous significantly upregulated and downregulated DEGs in the volcano plot.

A similar pattern was observed in vascular invasion-negative PDAC tissues compared with normal pancreatic tissues (Figure 1C,D), indicating widespread transcriptional alterations in PDAC irrespective of vascular invasion status.

To further identify vascular invasion-associated molecular features, we compared the DEG sets from these two analyses using a Venn diagram (Figure 1E). A large proportion of DEGs were shared between the two PDAC subgroups, including 5395 commonly upregulated genes and 552 commonly downregulated genes, consistent with core transcriptional alterations in PDAC. Based on the predefined subgroup-specific screening criteria, we retained 140 genes that were uniquely upregulated in vascular invasion-positive tumors compared with normal pancreas and 32 genes that were uniquely downregulated in vascular invasion-negative tumors compared with normal pancreas. These 172 subgroup-specific dysregulated genes were considered vascular invasion-associated candidate genes for downstream analyses and are provided in Supplementary Data S3. This strategy was intended to enrich candidate genes preferentially associated with vascular invasion-related transcriptional dysregulation while reducing the influence of transcriptional changes shared by both PDAC subgroups. To further clarify angiogenesis-related alterations in the nonvascular phenotype, we summarized angiogenesis-related genes differentially expressed in vascular invasion-negative PDAC tissues compared with normal pancreas in Supplementary Data S4. To visualize these changes and assess potential redundancy among angiogenesis-related regulators, we additionally plotted angiogenesis-related genes across the vascular invasion-positive and vascular invasion-negative comparisons, highlighting genes altered in the nonvascular phenotype in Supplementary Figure S1. These results indicate that angiogenesis-related transcriptional remodeling may also occur in vascular invasion-negative tumors and should be interpreted as part of broader PDAC-associated vascular remodeling rather than as a feature exclusively restricted to vascular invasion-positive tumors.

Functional annotation of these 172 genes was then performed. KEGG pathway enrichment analysis using KOBAS showed significant enrichment in pathways mainly related to glycan biosynthesis and degradation, amino sugar and nucleotide sugar metabolism, the pentose phosphate pathway, glycolysis and gluconeogenesis, and other metabolic processes (Figure 1F). Consistently, integrated enrichment analysis using Metascape highlighted terms associated with non-coding RNA (ncRNA) metabolic processes, translation, lysosome, nucleotide excision repair, RNA modification and transcription-related processes, and protein processing pathways (Figure 1G). Together, these results indicate that vascular invasion-associated candidate genes in PDAC are enriched in metabolic and macromolecule-processing programs, providing a basis for subsequent prognostic modeling and functional investigation.

### 3.3. Construction and Validation of a Vascular Invasion-Related Prognostic Model in Pancreatic Ductal Adenocarcinoma

**Figure 2:**
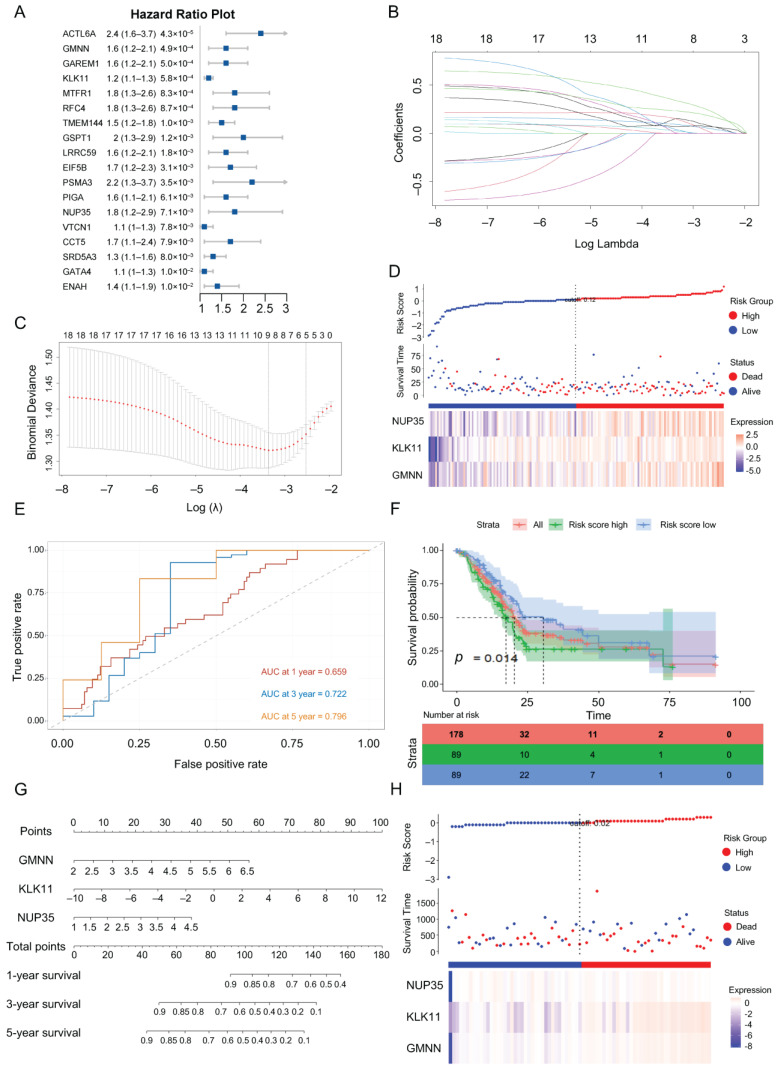
Construction and external validation of a vascular invasion-related prognostic signature in PDAC. (**A**) Forest plot of univariate Cox regression analysis for vascular invasion-associated genes in TCGA-PAAD. (**B**) LASSO Cox coefficient profiles for candidate prognostic genes. Each colored trace represents the coefficient trajectory of one candidate gene. (**C**) Tenfold cross-validation curve for selection of the optimal penalty parameter (λ) in the LASSO model. In panels B and C, the numbers on the top x-axis indicate the number of genes with nonzero coefficients at the corresponding λ values. Repeated numbers indicate the same number of retained genes across adjacent λ values. (**D**) Risk score distribution, survival status, and expression heatmap of the three-gene signature (NUP35, GMNN, and KLK11) in TCGA-PAAD. (**E**) Time-dependent receiver operating characteristic (ROC) curves and area under the curve (AUC) values for 1-, 3-, and 5-year overall survival (OS) prediction in TCGA-PAAD. (**F**) Kaplan–Meier OS curves stratified by the median risk score in TCGA-PAAD. (**G**) Nomogram based on the three-gene signature for predicting 1-, 3-, and 5-year OS. The GMNN, KLK11, and NUP35 axes represent mRNA expression levels used in the model. “Points” indicates the unitless point score assigned to each gene according to its expression level, and “Total points” represents the sum of the three gene-specific point scores. The 1-, 3-, and 5-year survival axes indicate the estimated OS probabilities corresponding to the total points. (**H**) Risk score distribution, survival status, and signature gene expression in the ICGC PACA-AU validation cohort.

To construct a vascular invasion-related prognostic model for PDAC, we first performed univariate Cox regression analysis on the candidate genes and identified 18 genes significantly associated with OS (Figure 2A). Several genes showed significant associations with poor prognosis, supporting their potential contribution to survival stratification.

To reduce overfitting and optimize model performance, LASSO Cox regression was applied. The coefficient trajectories and tenfold cross-validation are shown in Figure 2B,C, respectively, and the optimal penalty parameter (λ) was selected accordingly. Through this sequential screening strategy, NUP35, GMNN, and KLK11 were selected as the final prognostic genes and were used to construct the vascular invasion-related signature. Based on the coefficients derived from the multivariable Cox model, the final risk score formula was: Risk score = 0.4244379 × (GMNN mRNA expression) + 0.1527611 × (KLK11 mRNA expression) + 0.3661965 × (NUP35 mRNA expression).

Using the median risk score as the cutoff, patients in the TCGA cohort were divided into high-risk and low-risk groups. The distributions of risk score, survival status, and expression patterns of the three signature genes are shown in Figure 2D. Compared with the low-risk group, the high-risk group showed higher risk scores, a greater proportion of death events, and distinct expression profiles of the three-gene signature.

The prognostic performance of the model was evaluated using time-dependent ROC analysis. As shown in Figure 2E, the model achieved AUC values of 0.659, 0.722, and 0.796 for predicting 1-, 3-, and 5-year OS, respectively, indicating good predictive performance. Consistently, Kaplan–Meier survival analysis demonstrated significantly poorer OS in the high-risk group than in the low-risk group (Figure 2F, log-rank *p* = 0.014). To facilitate individualized survival prediction, a nomogram incorporating the three-gene signature was constructed (Figure 2G), providing a quantitative tool for estimating 1-, 3-, and 5-year survival probabilities. To further assess whether the three-gene signature yielding prognostic information independent of available clinicopathological variables, we performed multivariable Cox regression analysis incorporating the risk score, age, gender, and simplified pathological stage. Because the number of stage III/IV cases was small, pathological stage was collapsed into stage I and stage II–IV groups for model stability. The risk score remained independently associated with OS after adjustment for these covariates (HR = 2.727, 95% confidence interval (CI), 1.659–4.481, *p* = 7.58 × 10^−5^; Supplementary Data S5).

External validation was then performed in the independent PACA-AU cohort. The distributions of risk score, survival status, and expression patterns of the three signature genes in the validation cohort (Figure 2H) showed trends generally consistent with those observed in the TCGA cohort, supporting the reproducibility of the vascular invasion-related prognostic model across independent patient populations.

### 3.4. Functional Characterization of Differentially Expressed Genes Between High- and Low-Risk Groups

**Figure 3:**
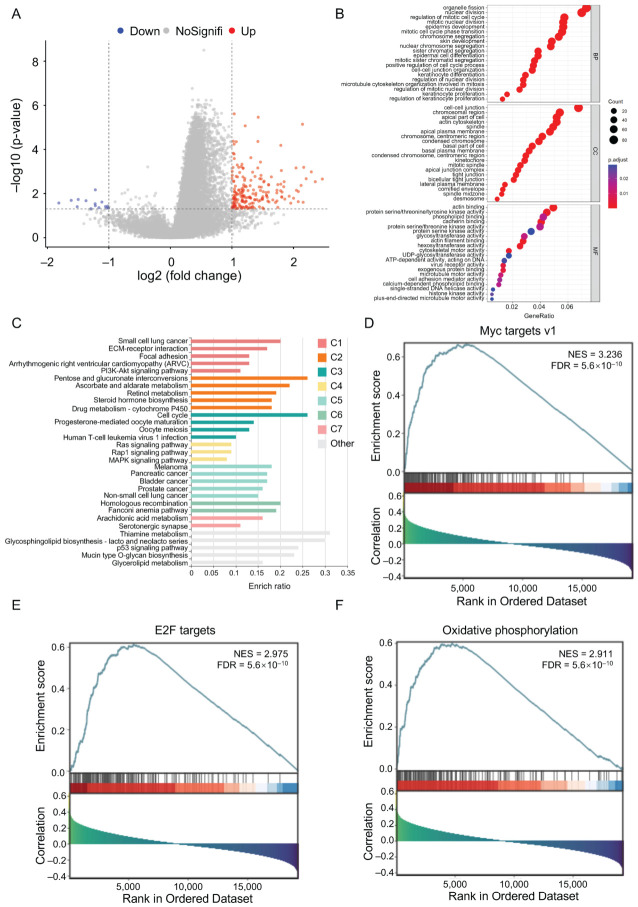
Transcriptomic differences between high- and low-risk PDAC groups. (**A**) Volcano plot of DEGs between high- and low-risk groups. “Up”, “Down”, and “NoSignifi” indicate significantly upregulated, significantly downregulated, and nonsignificant genes, respectively. (**B**) GO enrichment analysis (BP/CC/MF) of DEGs between risk groups. p.adjust indicates the multiple-testing adjusted *p* value. GeneRatio indicates the proportion of input genes annotated to a given Gene Ontology term. (**C**) KEGG enrichment analysis of DEGs between risk groups. Enrich ratio indicates the proportion of input genes mapped to each KEGG pathway. (**D**–**F**) Representative GSEA plots for hallmark pathways, including MYC targets v1 (HALLMARK_MYC_TARGETS_V1, a standard MSigDB Hallmark gene set of MYC target genes; (**D**), E2F targets (**E**), and oxidative phosphorylation (**F**). NES and FDR values are shown in each GSEA plot. The enrichment curve represents the running enrichment score, and the “Correlation” track represents the ranked list metric used for GSEA ranking rather than pairwise gene–gene correlation. BP, biological process; CC, cellular component; FDR, false discovery rate; MF, molecular function; NES, normalized enrichment score.

To investigate the biological differences underlying the prognostic risk stratification, we performed differential expression analysis between the high- and low-risk groups defined by the vascular invasion-related signature using a quartile-based cutoff. As shown in Figure 3A, the volcano plot identified a substantial number of DEGs, including both upregulated and downregulated genes between the two risk groups.

GO enrichment analysis revealed that these DEGs were significantly enriched in biological processes and cellular functions related to cell cycle progression and mitotic activity, including organelle fission, nuclear division, mitotic cell cycle, and chromosome segregation (Figure 3B). Enriched cellular component terms were mainly associated with chromosomal regions, spindle- and microtubule-related structures, and cell junction-associated compartments, while molecular function terms included protein kinase binding, actin and cytoskeletal binding, and microtubule motor activity (Figure 3B).

Consistently, KEGG pathway enrichment analysis showed significant enrichment of multiple cancer-related pathways, including extracellular matrix (ECM)–receptor interaction, focal adhesion, phosphoinositide 3-kinase (PI3K)–protein kinase B (Akt) signaling, cell cycle, rat sarcoma virus (Ras), Ras-related protein 1 (Rap1), mitogen-activated protein kinase (MAPK) signaling, p53 signaling, and pancreatic cancer pathways (Figure 3C), supporting marked molecular differences between the two risk strata.

Furthermore, GSEA identified significant enrichment of hallmark pathways represented by MYC proto-oncogene (MYC) targets v1 (HALLMARK_MYC_TARGETS_V1, a standard MSigDB Hallmark gene set of MYC target genes; Figure 3D), E2F targets (Figure 3E), and oxidative phosphorylation (Figure 3F), indicating distinct proliferative and metabolic programs associated with the risk signature. Collectively, these results suggest that the vascular invasion-related prognostic model captures biologically distinct PDAC subgroups characterized by differences in proliferation-related signaling, tumor-associated pathways, and metabolic regulation.

### 3.5. Association of the Three Signature Genes with Survival and Angiogenesis-Related Features

**Figure 4:**
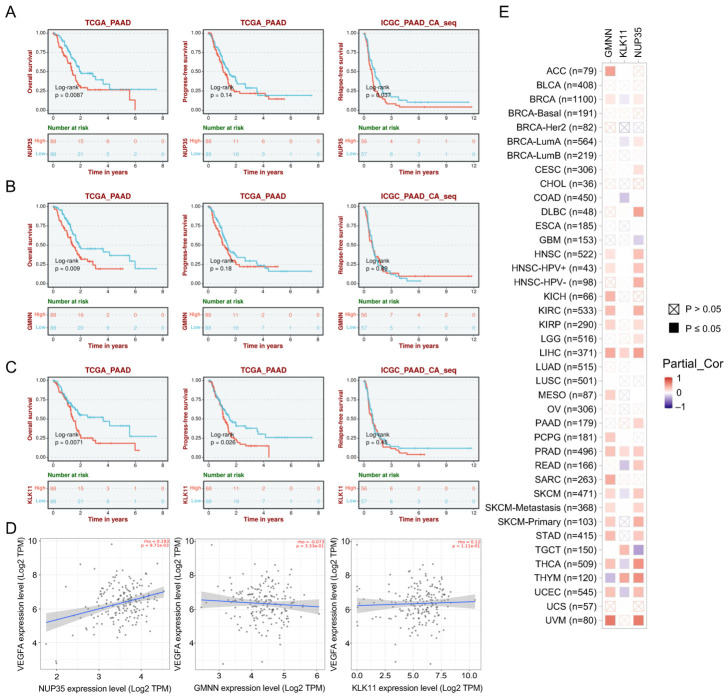
Prognostic relevance of signature genes and association with VEGFA. (**A**–**C**) Kaplan–Meier survival analyses for NUP35 (**A**), GMNN (**B**), and KLK11 (**C**) in TCGA and ICGC cohorts for available endpoints, including overall survival (OS), progression-free survival (PFS), and relapse-free survival (RFS), as indicated. In panels (**A**–**C**), the red and blue curves represent the high- and low-expression groups of the indicated gene, respectively. Expression values are shown as transcripts per million (TPM), where applicable. (**D**) Correlation between signature gene expression (NUP35, GMNN, and KLK11) and VEGFA expression in PDAC. “rho” indicates Spearman’s rank correlation coefficient. The blue line represents the fitted correlation trend, and the gray shading represents the 95% confidence interval around the fitted line. (**E**) Pan-cancer correlation heatmap showing associations between the three signature genes and VEGFA-related features across tumor types. “Partial_Cor” indicates the partial correlation coefficient adjusted for tumor purity.

To further evaluate the clinical relevance of the three signature genes (NUP35, GMNN, and KLK11), survival analyses were performed in the TCGA and ICGC cohorts (Figure 4A–C). In the TCGA-PAAD cohort, higher expression of all three genes was associated with significantly worse OS (NUP35, *p* = 0.0087; GMNN, *p* = 0.009; KLK11, *p* = 0.0071). For progression-related endpoints, KLK11 was significantly associated with worse PFS in TCGA (*p* = 0.026), whereas the associations for NUP35 (*p* = 0.14) and GMNN (*p* = 0.18) did not reach statistical significance. In the ICGC PACA-AU cohort, NUP35 expression was associated with poorer RFS (*p* = 0.037), whereas GMNN (*p* = 0.89) and KLK11 (*p* = 0.45) were not significantly associated with this endpoint.

Because angiogenesis is closely linked to vascular invasion, we next examined the correlation between the three signature genes and VEGFA expression in PDAC (Figure 4D). Among the three genes, NUP35 showed a significant positive correlation with VEGFA expression (Spearman’s rho = 0.193, *p* = 9.71 × 10^−3^), whereas GMNN and KLK11 did not show significant correlations in this cohort.

To further assess the broader relevance of these associations, we performed pan-cancer correlation analysis (Figure 4E), which showed heterogeneous correlation patterns across tumor types and suggested that the relationships between the three signature genes and angiogenesis-related features may be context dependent. Notably, the PDAC findings highlighted NUP35 as the gene most consistently linked to both adverse prognosis and VEGFA-associated signaling, supporting its prioritization for subsequent analyses.

### 3.6. Functional Characterization of NUP35 in Pancreatic Cancer

**Figure 5:**
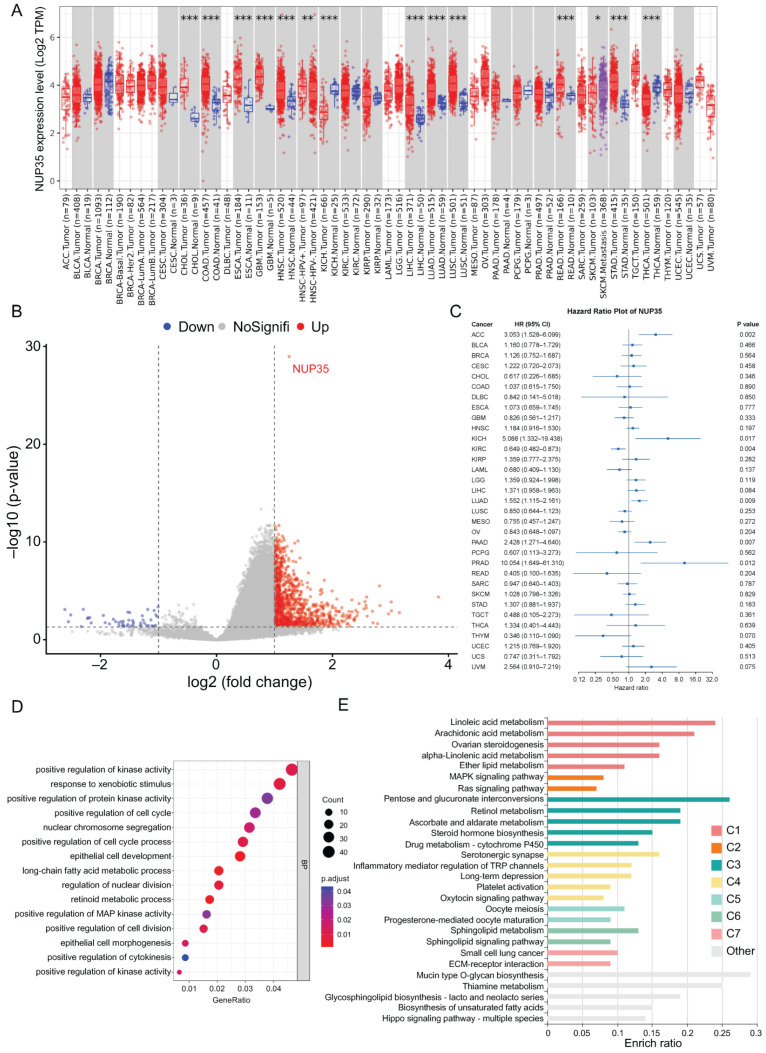
Pan-cancer characterization and functional annotation of NUP35-associated transcriptomic features. (**A**) Pan-cancer expression profile of NUP35 across TCGA tumor and normal tissues. Colors indicate sample types as labeled on the x-axis. (**B**) Volcano plot of DEGs between high- and low-NUP35 expression groups in PDAC. “Up”, “Down”, and “NoSignifi” indicate significantly upregulated, significantly downregulated, and nonsignificant genes, respectively. (**C**) Pan-cancer Cox regression (forest plot) showing associations between NUP35 expression and survival across tumor types. (**D**) GO enrichment analysis of NUP35-associated DEGs in PDAC. p.adjust indicates the multiple-testing adjusted *p* value. GeneRatio indicates the proportion of input genes annotated to a given Gene Ontology term. (**E**) KEGG enrichment analysis of NUP35-associated DEGs in PDAC. Enrich ratio indicates the proportion of input genes mapped to each KEGG pathway. TPM, transcripts per million; HR, hazard ratio; CI, confidence interval; BP, biological process. * *p* < 0.05; ** *p* < 0.01; *** *p* < 0.001.

Among the three signature genes, NUP35 was prioritized for further investigation based on its association with adverse prognosis and its positive correlation with VEGFA expression in PDAC. Pan-cancer expression analysis showed that NUP35 was differentially expressed across multiple tumor types with aberrant upregulation observed in several cancers, including PAAD (Figure 5A), suggesting a broader role in tumor biology.

To further investigate the molecular features associated with NUP35 in pancreatic cancer, PDAC samples were stratified into high- and low-NUP35 expression groups followed by differential expression analysis. As shown in Figure 5B, the volcano plot identified numerous DEGs between the two groups, indicating substantial transcriptomic differences associated with NUP35 expression status.

Pan-cancer survival analysis using a Cox proportional hazards framework further demonstrated that elevated NUP35 expression was associated with poor prognosis in multiple malignancies, including PAAD (Figure 5C), supporting its potential clinical relevance across cancer types.

Functional enrichment analysis of NUP35-associated DEGs revealed significant GO terms related to positive regulation of kinase activity, cell cycle and cell division processes, nuclear chromosome segregation, and MAPK activity, as well as epithelial development-related processes (Figure 5D). KEGG pathway analysis further highlighted enrichment in pathways associated with MAPK signaling, Ras signaling, ECM–receptor interaction, sphingolipid signaling and metabolism, and multiple metabolic pathways (Figure 5E). Together, these findings suggest that NUP35 is associated with transcriptional programs linked to proliferative signaling, tumor progression, and microenvironment-related pathways, providing a rationale for subsequent functional validation in PDAC cells.

### 3.7. NUP35 Promotes Proliferation, Migration, and Invasion in Pancreatic Cancer Cells

**Figure 6:**
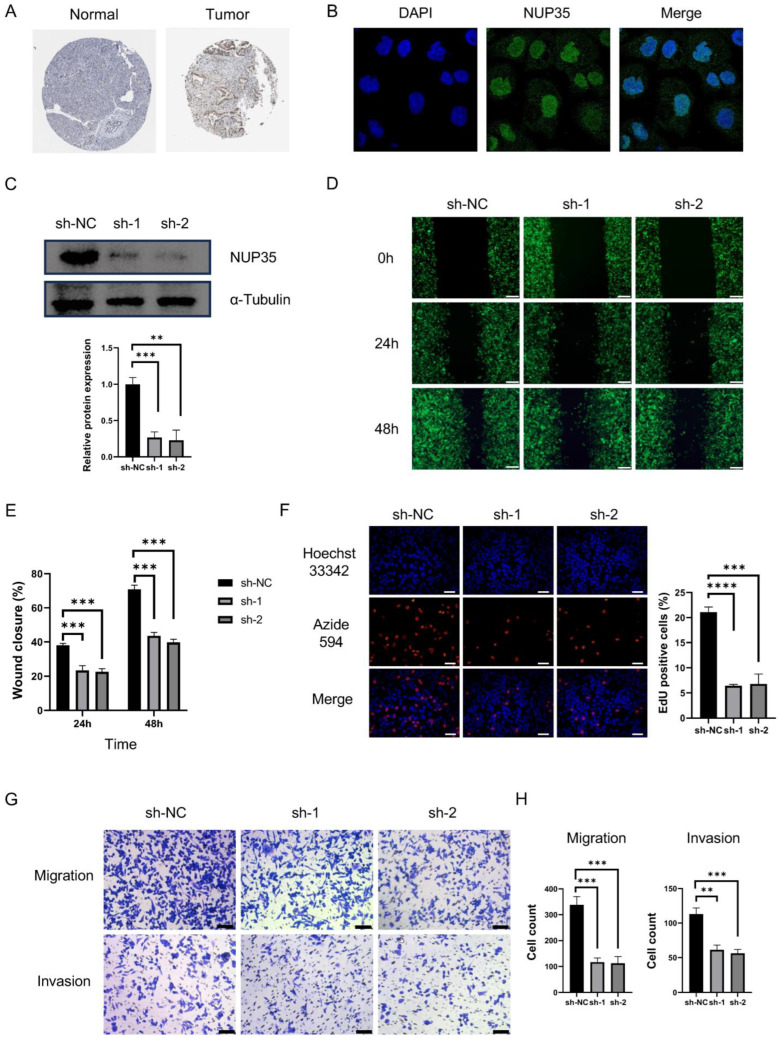
NUP35 promotes proliferation, migration, and invasion of pancreatic cancer cells. (**A**) Representative HPA IHC images showing NUP35 protein expression in normal pancreatic tissues and pancreatic cancer. (**B**) Representative HPA IF images showing subcellular localization of NUP35. (**C**) Western blotting showing NUP35 knockdown efficiency in PATU 8988 cells (sh-NC, sh-1, and sh-2) and corresponding quantification. (**D**) Representative wound healing images at indicated time points after NUP35 knockdown. (**E**) Quantification of wound closure in D. (**F**) Representative EdU staining images and quantification of EdU-positive cells after NUP35 knockdown. (**G**) Representative transwell images for migration and invasion assays. (**H**) Quantification of migrated and invaded cells in G. Data are presented as mean ± SD from at least three independent biological replicates. Statistical significance was assessed by one-way ANOVA with appropriate post hoc testing. ** *p* < 0.01; *** *p* < 0.001; **** *p* < 0.0001.

To further characterize NUP35 at the protein level, we examined its expression and subcellular localization using the HPA. HPA IHC images showed higher NUP35 protein expression in pancreatic cancer tissues than in normal pancreatic tissues (Figure 6A). HPA IF images further indicated that NUP35 was mainly localized to the nuclear region (Figure 6B), consistent with its role as a nucleoporin-associated protein.

To determine the functional role of NUP35 in pancreatic cancer cells, we established NUP35 knockdown models using two independent shRNAs (sh-1 and sh-2). Western blot analysis confirmed effective downregulation of NUP35 protein expression in both knockdown groups compared with the negative control (sh-NC) (Figure 6C).

Wound healing assays showed that NUP35 knockdown significantly impaired cell migration, with both sh-1 and sh-2 groups exhibiting reduced wound closure at 24 and 48 h relative to sh-NC cells (Figure 6D,E). Consistently, EdU incorporation assays demonstrated a marked reduction in proliferative activity following NUP35 downregulation (Figure 6F).

Furthermore, transwell assays showed that knockdown of NUP35 markedly suppressed both migratory and invasive capacities of pancreatic cancer cells, and these effects were consistently observed with both shRNAs (Figure 6G,H). Collectively, these findings suggest that NUP35 contributes to malignant phenotypes, including proliferation, migration, and invasion, in pancreatic cancer cells.

### 3.8. NUP35 Knockdown Attenuates Tumor-Induced Angiogenesis and ERK–VEGFA Signaling

**Figure 7:**
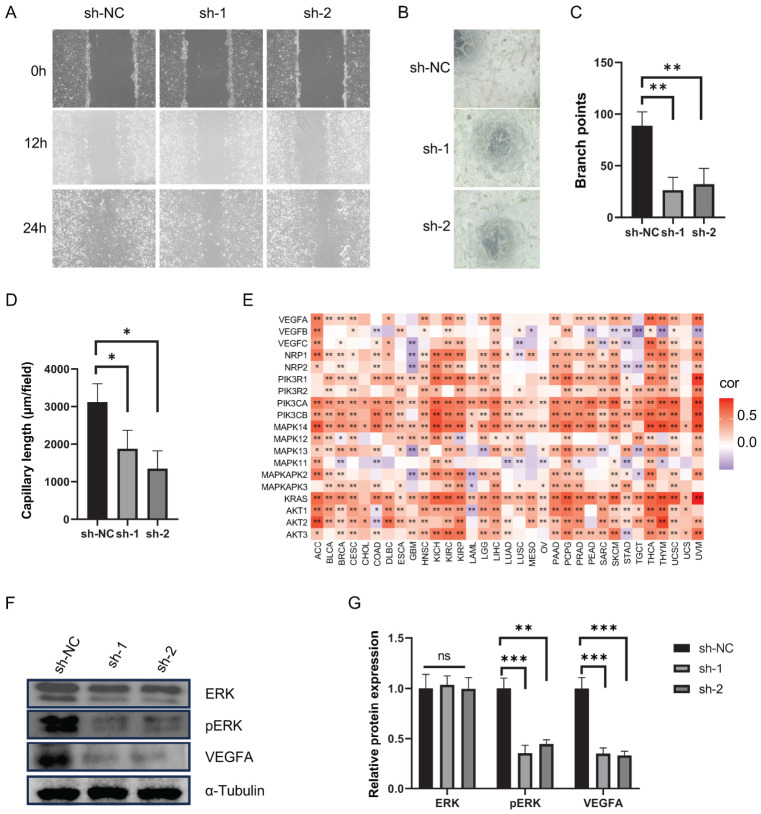
NUP35 enhances tumor-driven angiogenesis via ERK–VEGFA signaling. (**A**) Endothelial wound healing assay showing migration of HUVECs cultured with CM from sh-NC, sh-1, or sh-2 tumor cells at indicated time points. (**B**) Representative tube formation images of HUVECs cultured with CM from sh-NC, sh-1, or sh-2 tumor cells. (**C**) Quantification of branch points in tube formation assays. (**D**) Quantification of capillary length (μm/field) in tube formation assays. (**E**) Pan-cancer correlation heatmap showing associations between NUP35 expression and angiogenesis-related signaling genes across cancer types. “cor” indicates the correlation coefficient between NUP35 expression and the indicated genes. (**F**) Western blotting showing ERK, p-ERK, and VEGFA protein levels in NUP35-knockdown PATU 8988 cells. (**G**) Quantification of protein levels shown in F; p-ERK and VEGFA were normalized to α-tubulin. Data are presented as mean ± SD from at least three independent biological replicates. Statistical significance was assessed by one-way ANOVA with post hoc testing. ns, not significant; * *p* < 0.05; ** *p* < 0.01; *** *p* < 0.001.

To determine whether NUP35 regulates tumor-induced angiogenesis, endothelial cell assays were performed using conditioned medium collected from control (sh-NC) and NUP35-knockdown (sh-1 and sh-2) pancreatic cancer cells. In the wound healing assay, conditioned medium from NUP35-silenced cells significantly reduced endothelial cell migratory capacity, as evidenced by delayed wound closure compared with the sh-NC group (Figure 7A).

Consistently, tube formation assays showed that endothelial cells cultured with conditioned medium from NUP35-knockdown cells formed fewer and less developed capillary-like networks than those cultured with control conditioned medium (Figure 7B). Quantitative analysis further confirmed significant reductions in tube formation metrics in both sh-1 and sh-2 groups (Figure 7C,D), indicating impaired angiogenic potential after NUP35 silencing in tumor cells.

To further support the angiogenesis-related role of NUP35, pan-cancer correlation analysis was performed for NUP35 and genes involved in VEGF, PI3K, AKT, and MAPK signaling. As shown in Figure 7E, NUP35 expression was broadly and positively correlated with multiple angiogenesis-associated and downstream signaling genes across tumor types, including VEGFA/VEGFB/VEGFC, neuropilin 1 (NRP1) and neuropilin 2 (NRP2), PI3K family genes, MAPK pathway genes, KRAS, and AKT family genes.

Mechanistically, Western blot analysis of NUP35-knockdown PATU 8988 cell lysates showed that NUP35 knockdown markedly decreased p-ERK and VEGFA protein levels, while total ERK levels remained unchanged (Figure 7F,G). These findings suggest that NUP35 may contribute to tumor-induced angiogenic activity, at least in part, through regulation of ERK activation and VEGFA expression in pancreatic cancer cells.

### 3.9. Exploratory Pan-Cancer Analyses of NUP35

Finally, exploratory pan-cancer analyses were performed to provide broader biological context for NUP35 beyond PDAC. Because tumor angiogenesis and the immune microenvironment are closely interconnected, we examined whether NUP35 expression was associated with angiogenesis- and immune-related features across cancer types. NUP35 expression was positively correlated with VEGFA expression in several representative cancers, including breast invasive carcinoma (BRCA), thyroid carcinoma (THCA), head and neck squamous cell carcinoma (HNSC), kidney renal clear cell carcinoma (KIRC), and liver hepatocellular carcinoma (LIHC), suggesting a broader association with angiogenesis-related signaling (Supplementary Figure S2A). NUP35 expression also showed context-dependent correlations with TMB and MSI across different tumor types (Supplementary Figure S2B,C). In addition, NUP35 expression was associated with immune checkpoint molecules and immune cell infiltration estimates in a tumor type-dependent manner (Supplementary Figure S2D,E). Taken together, these exploratory pan-cancer findings suggest that NUP35 is broadly associated with angiogenesis-related signaling and immune microenvironment features and may be linked to tumor-promoting biological programs in a context-dependent manner.

## 4. Discussion

**Figure 8:**
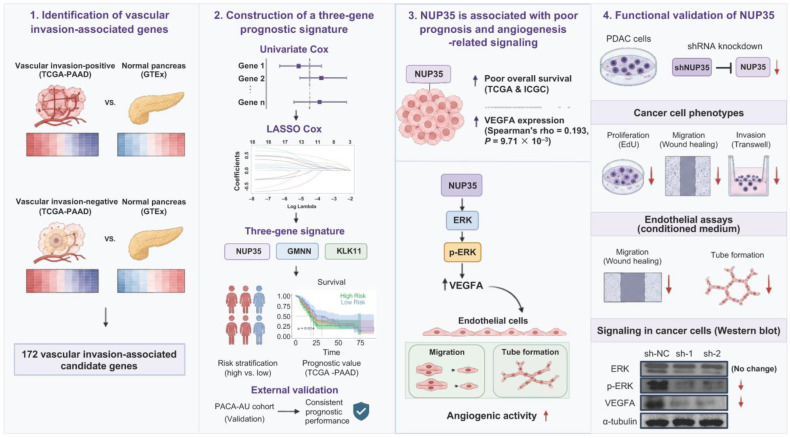
Schematic summary of the study design and proposed NUP35-related angiogenic mechanism in PDAC. Vascular invasion-associated candidate genes were identified from TCGA-PAAD and GTEx data, followed by construction and external validation of a three-gene prognostic signature comprising NUP35, GMNN, and KLK11. NUP35 was prioritized for functional validation based on its prognostic relevance and positive association with VEGFA. NUP35 knockdown reduced PDAC cell proliferation, migration, and invasion, attenuated endothelial migration and tube formation, and decreased p-ERK and VEGFA levels, supporting a putative NUP35-ERK-VEGFA axis in tumor-induced angiogenic activity. PDAC, pancreatic ductal adenocarcinoma.

PDAC remains one of the most aggressive malignancies, with poor long-term survival [1,16,17]. Vascular invasion is strongly associated with tumor dissemination and adverse outcomes [5], yet its molecular basis in PDAC is incompletely understood [10]. Here, we established a vascular invasion-oriented molecular framework, developed and externally validated a three-gene prognostic signature (NUP35, GMNN, and KLK11), and identified NUP35 as a candidate regulator of malignant phenotypes and tumor-induced angiogenic activity, at least in part through the ERK–VEGFA signaling axis. Pan-cancer analyses further linked NUP35 to angiogenesis- and immune-related features across tumor types. A schematic overview of the study design and proposed NUP35–ERK–VEGFA–angiogenesis axis is provided in Figure 8.

A key methodological feature of this work was the use of subgroup-specific filtering after comparing vascular invasion-positive and -negative PDAC tissues separately with normal pancreas, thereby reducing confounding from transcriptomic changes shared across PDAC. Functional annotation highlighted metabolic and macromolecule-processing programs (Figure 1F,G), suggesting that vascular invasion may involve broader metabolic and biosynthetic reprogramming in addition to established invasion-associated pathways, consistent with evidence linking metabolic remodeling to tumor invasion and progression [18,19,20].

Using the resulting gene set, we derived a parsimonious three-gene model with favorable prognostic performance in TCGA (Figure 2A–F) and consistent trends in the PACA-AU cohort (Figure 2H). Moreover, multivariable Cox analysis incorporating age, gender, and pathological stage further supported the independent prognostic value of the risk score. Transcriptomic analyses between risk strata revealed enrichment of cell cycle progression (Figure 3B), ECM–receptor interaction, focal adhesion, PI3K-Akt/MAPK signaling (Figure 3C), and hallmark proliferative programs (MYC and E2F targets) (Figure 3D,E), supporting that the signature captures biologically distinct PDAC subgroups rather than serving only as a statistical classifier. These programs align with well-established drivers of PDAC aggressiveness and progression [21,22,23].

Established clinicopathological predictors, including TNM stage, nodal status, tumor grade, resection margin status, carbohydrate antigen 19-9 (CA19-9) level, and treatment modality, remain essential for prognostic assessment in PDAC [24]. The present three-gene model was developed from public transcriptomic datasets and should be interpreted as an exploratory molecular signature that may complement, rather than replace, conventional clinical predictors. Because detailed treatment information, resection status, margin status, and CA19-9 levels were incomplete or inconsistently available in the public datasets, comprehensive comparison with all established predictors could not be reliably performed. Future validation using clinically annotated PDAC cohorts and tumor samples is needed to determine the added prognostic value and clinical applicability of this signature.

Among the three genes, NUP35 was prioritized based on more consistent prognostic associations across cohorts (Figure 4A–C) and its significant positive correlation with VEGFA in PDAC (Figure 4D). NUP35, a nucleoporin-associated protein involved in nuclear pore complex architecture [25,26], has been insufficiently characterized in PDAC. Our analyses linked NUP35 to kinase-regulatory, MAPK-related, cell cycle, and microenvironment-associated pathways, consistent with evidence that nucleoporins can influence oncogenic signaling programs in cancer [27,28,29]. It should be noted that the enrichment analysis based on NUP35 expression quartiles in TCGA-PAAD reflects bulk-tumor transcriptomic associations rather than direct molecular consequences of experimental NUP35 knockdown. Therefore, although the enrichment results mainly highlighted proliferation-, cell cycle-, MAPK-, and ECM-related programs (Figure 5D,E), the angiogenesis-related role of NUP35 was inferred from additional evidence, including VEGFA correlation analysis (Figure 4D), reduced VEGFA and p-ERK protein levels after NUP35 silencing (Figure 7F,G), and functional endothelial assays using conditioned medium (Figure 7A–D). Functionally, NUP35 knockdown suppressed PDAC cell proliferation, migration, and invasion (Figure 6D–H), and conditioned medium from NUP35-silenced cells impaired endothelial migration and tube formation (Figure 7A–D), indicating reduced tumor-driven angiogenic activity.

Mechanistically, NUP35 silencing decreased p-ERK and VEGFA protein levels without altering total ERK, suggesting that NUP35 may enhance ERK activation and downstream VEGFA expression (Figure 7F,G). Given the central role of ERK/MAPK signaling in PDAC progression [21,30] and angiogenic regulation [31,32], these findings support a putative NUP35-ERK-VEGFA-angiogenesis axis in PDAC. In this model, NUP35 may act upstream of ERK activation in pancreatic cancer cells, thereby promoting VEGFA expression and enhancing the pro-angiogenic activity of tumor-cell conditioned medium toward endothelial cells. This axis provides a potential link between a nucleoporin-associated protein and tumor-vascular crosstalk, extending the possible biological relevance of nucleoporins beyond nuclear transport and gene regulation. However, the current evidence is mainly based on loss-of-function experiments and the upstream mechanism by which NUP35 regulates ERK phosphorylation remains to be clarified. In addition, endothelial VEGFR signaling and other VEGF family members were not directly assessed in the present study. Therefore, future studies are needed to determine whether additional VEGF ligands or receptors participate in NUP35-regulated tumor-endothelial communication.

Finally, pan-cancer analyses showed that NUP35 is associated with VEGFA and VEGF-, PI3K-, AKT-, and MAPK-related genes in multiple tumors (Figure 7E and Supplementary Figure S2A) and displays context-dependent associations with TMB and MSI, immune checkpoint molecules, and immune infiltration signatures (Supplementary Figure S2B–E). The rationale for including immune-related analyses was based on the recognized crosstalk between angiogenesis and the immune microenvironment, whereby abnormal tumor vasculature may shape immune cell infiltration and immune exclusion [33]. Nevertheless, these immune-related analyses were exploratory and should not be interpreted as direct mechanistic evidence for NUP35-regulated angiogenesis. While correlation-based, these results suggest that NUP35 may mark tumor-promoting angiogenesis–immune states in certain cancer contexts.

In addition to NUP35, the other two signature genes, GMNN and KLK11, may provide complementary biological information within the three-gene signature. GMNN encodes geminin, a key regulator of DNA replication licensing that prevents re-replication and contributes to genomic stability during cell cycle progression [34]. Because dysregulated DNA replication and cell cycle activation are central features of aggressive tumor growth, increased GMNN expression may reflect the proliferative component captured by the risk model. Consistent with this interpretation, our risk-stratified transcriptomic analyses showed enrichment of cell cycle (Figure 3B), MYC (Figure 3D), and E2F-related programs (Figure 3E). Beyond its role in proliferation, geminin has also been implicated in metastatic phenotypes in a context-dependent manner, suggesting that GMNN may contribute to malignant progression programs [35].

KLK11, a member of the kallikrein-related peptidase family, represents a different biological dimension of the signature [36]. Kallikrein-related peptidases are secreted or extracellular proteases involved in proteolytic remodeling, biomarker biology, and tumor–microenvironment interactions [37]. KLK11 has been associated with prognosis in several malignancies and may influence growth factor availability, for example, through degradation of insulin-like growth factor-binding protein 3 (IGFBP-3) and release of insulin-like growth factor activity [38]. Therefore, KLK11 may capture protease-associated or microenvironment-related aspects of PDAC aggressiveness. Together, GMNN, and KLK11 likely complement NUP35 by representing proliferative, extracellular, and microenvironmental components of the vascular invasion-related prognostic signature, whereas NUP35 was prioritized for functional validation because of its stronger association with VEGFA and angiogenesis-related features in PDAC.

Several limitations should be acknowledged. First, the model was developed from retrospective public datasets and although external validation and multivariable Cox analysis were performed to reduce overfitting and assess independence, prospective validation in larger clinically annotated cohorts remains necessary. Because TCGA-PAAD contains only a limited number of normal pancreatic samples, GTEx normal pancreas data were incorporated after batch-effect correction. Therefore, future studies using matched tumor and adjacent normal tissues would further strengthen the robustness of the signature. Second, some clinically important variables including resection margin status, CA19-9 levels, detailed treatment information, and standardized vascular invasion annotation were incomplete in the public datasets, limiting comprehensive comparison with all established clinical predictors. Third, functional validation was mainly based on in vitro loss-of-function assays. Although two independent shRNAs consistently supported a role for NUP35 in malignant and pro-angiogenic phenotypes, rescue experiments, in vivo models, endothelial VEGFR signaling analysis, and pharmacological studies are needed to further define causality, downstream mechanisms, and therapeutic applicability. Finally, the pan-cancer analyses were exploratory and hypothesis-generating.

In summary, we propose a vascular invasion-related prognostic signature for PDAC and uncover NUP35 as a key regulator of malignant progression and tumor-induced angiogenesis, at least in part via ERK-VEGFA signaling, supporting its potential as a biomarker and highlighting NUP35-related pro-angiogenic signaling as a candidate therapeutic vulnerability requiring further investigation.

## Figures and Tables

**Table 1:** Clinicopathological characteristics of PDAC patients stratified by vascular invasion status.

Characteristics		Vascular Invasion-Positive (*n* = 104)	Vascular Invasion-Negative (*n* = 60)	*p* Value
age (years)		66.02 ± 10.84	64.83 ± 11.26	0.5114
gender (*n*)	Female	45	27	0.9104
	Male	53	33	
race (*n*)	Asian	8	3	0.4747
	Black or African American	4	1	
	White	83	56	
tumor size (cm)		3.92 ± 1.975	3.655 ± 1.454	0.753
tumor stage (*n*)	I	7	12	0.0311
	II	87	45	
	III + IV	2	3	
T stage (*n*)	T1 + T2	12	17	0.0189
	T3 + T4	85	43	
N stage (*n*)	N0	12	28	<0.0001
	N1	82	32	
M stage (*n*)	M0	43	23	0.957
	M1	2	1	
	MX	53	36	
tumor grade (*n*)	G1 + G2	38	30	0.2762
	G3 + G4	40	20	
tumor location (*n*)	Body	8	4	0.9373
	Head	63	38	
	Tail	9	6	
KRAS (*n*)	No mutated	6	3	0.7148
	Mutated	72	47	

## Data Availability

The datasets analyzed in this study are publicly available. TCGA-PAAD RNA-seq expression data and corresponding clinical information were downloaded from the GDC. Normal pancreas expression data were obtained from GTEx via the UCSC Xena platform. The PACA-AU cohort was accessed from the ICGC data portal. HPA IHC and IF images were retrieved from the HPA database. All other data supporting the findings of this study are available from the corresponding author upon reasonable request.

## Supplementary Materials

The following supporting information can be downloaded at: https://www.mdpi.com/article/10.3390/biomedicines14061253/s1. Figure S1: Angiogenesis-related gene alterations in vascular invasion-related PDAC phenotypes; Figure S2: Pan-cancer analyses link NUP35 to angiogenesis- and immune-related features. Data S1: Differential expression results for vascular invasion-positive PDAC tissues versus normal pancreas; Data S2: Differential expression results for vascular invasion-negative PDAC tissues versus normal pancreas; Data S3: Vascular invasion-associated candidate genes retained for downstream analyses; Data S4: Angiogenesis-related genes differentially expressed in vascular invasion-negative PDAC tissues compared with normal pancreas; Data S5: Multivariable Cox regression analysis of the three-gene risk score and available clinicopathological variables in TCGA-PAAD.
