# Supplementary material for: A Vascular Invasion-Related Gene Signature Identifies NUP35 as a Driver of Angiogenesis and Poor Prognosis in Pancreatic Ductal Adenocarcinoma
Source: Biomedicines. 2026 May 30;14(6):1253. doi: 10.3390/biomedicines14061253 (PMC13297405; doi:10.3390/biomedicines14061253)
Supplement: Supplementary file 1 [file biomedicines-14-01253-s001.zip › supplementary figure.pdf]

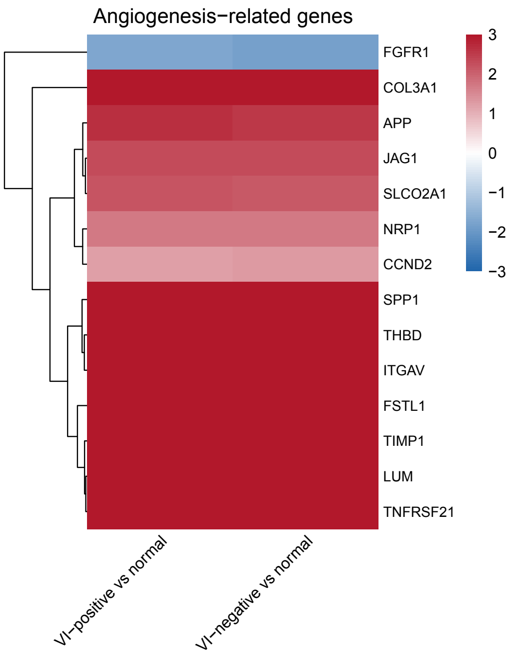

Supplementary Figure S1. Angiogenesis-related gene alterations in vascular invasion-related PDAC phenotypes. Heatmap showing log2 fold changes of angiogenesis-related genes in vascular invasion-positive and vascular invasion-negative PDAC tissues compared with normal pancreas. Genes shown were significantly altered in the vascular invasion-negative phenotype. Red indicates upregulation and blue indicates downregulation.

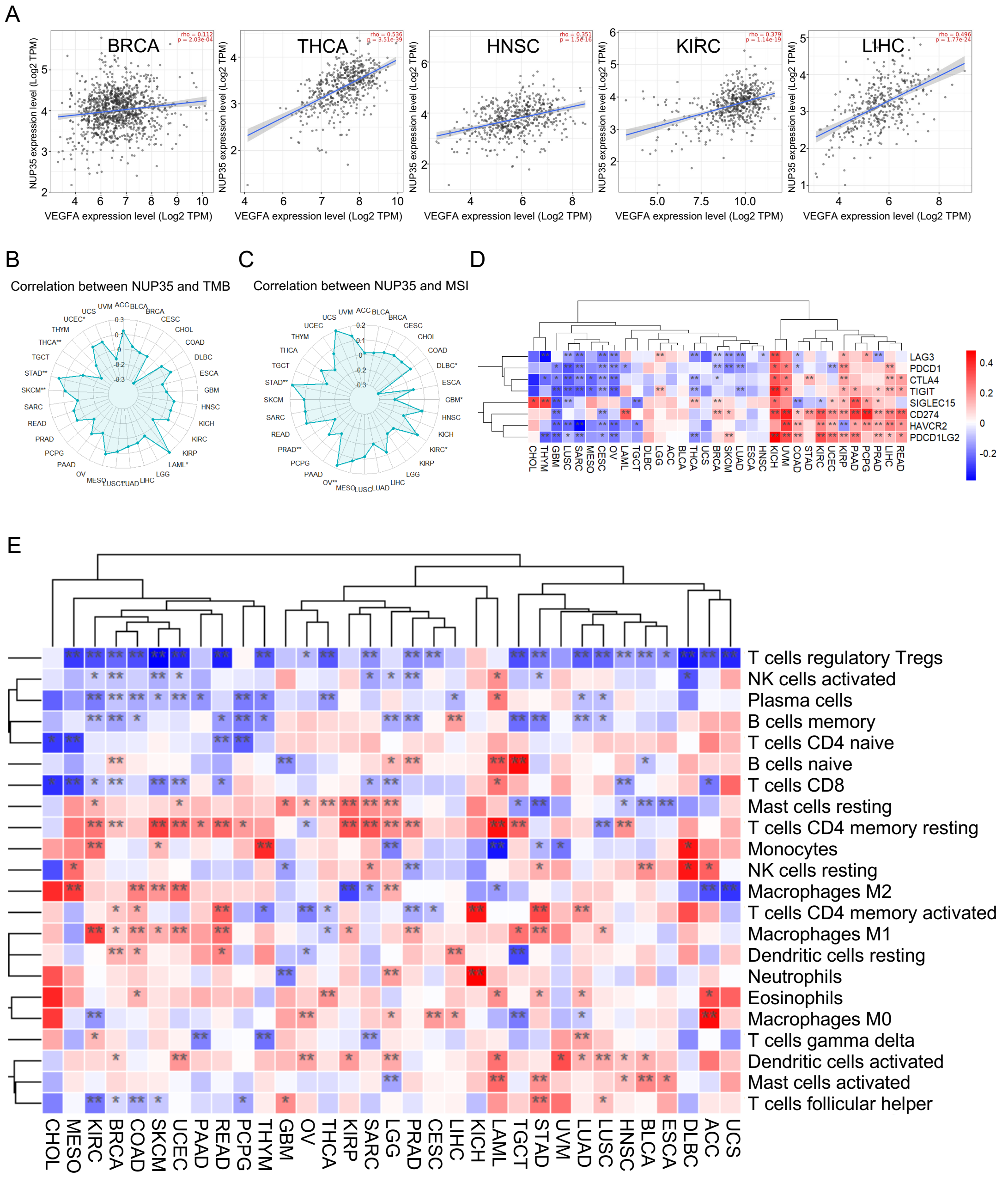

Supplementary Figure S2. Pan-cancer analyses link NUP35 to angiogenesis- and immune-related features. (A) Representative correlations between NUP35 and VEGFA expression in selected tumor types. (B) Pan-cancer correlations between NUP35 expression and TMB. (C) Pan-cancer correlations between NUP35 expression and MSI. (D) Pan-cancer correlation heatmap between NUP35 and immune checkpoint molecules. (E) Pan-cancer correlation heatmap between NUP35 and immune cell infiltration signatures across tumor types. TMB, tumor mutation burden; MSI, microsatellite instability.
